# Transcriptome Analysis of H_2_O_2_-Treated Wheat Seedlings Reveals a H_2_O_2_-Responsive Fatty Acid Desaturase Gene Participating in Powdery Mildew Resistance

**DOI:** 10.1371/journal.pone.0028810

**Published:** 2011-12-12

**Authors:** Aili Li, Rongzhi Zhang, Lei Pan, Lichuan Tang, Guangyao Zhao, Mingzhu Zhu, Jinfang Chu, Xiaohong Sun, Bo Wei, Xiangqi Zhang, Jizeng Jia, Long Mao

**Affiliations:** 1 National Key Facility for Crop Gene Resources and Genetic Improvement, Institute of Crop Science, MOA Key Lab for Germplasm and Biotechnology, Chinese Academy of Agricultural Sciences (CAAS), Beijing, People's Republic of China; 2 State Key Laboratory of Plant Genomics, National Centre for Plant Gene Research, Institute of Genetics and Developmental Biology, Chinese Academy of Sciences, Beijing, People's Republic of China; 3 The State Key Laboratory of Plant Cell and Chromosome Engineering, Institute of Genetics and Developmental Biology, Chinese Academy of Sciences, Beijing, People's Republic of China; Saint Louis University, United States of America

## Abstract

Hydrogen peroxide (H_2_O_2_) plays important roles in plant biotic and abiotic stress responses. However, the effect of H_2_O_2_ stress on the bread wheat transcriptome is still lacking. To investigate the cellular and metabolic responses triggered by H_2_O_2_, we performed an mRNA tag analysis of wheat seedlings under 10 mM H_2_O_2_ treatment for 6 hour in one powdery mildew (PM) resistant (PmA) and two susceptible (Cha and Han) lines. In total, 6,156, 6,875 and 3,276 transcripts were found to be differentially expressed in PmA, Han and Cha respectively. Among them, 260 genes exhibited consistent expression patterns in all three wheat lines and may represent a subset of basal H_2_O_2_ responsive genes that were associated with cell defense, signal transduction, photosynthesis, carbohydrate metabolism, lipid metabolism, redox homeostasis, and transport. Among genes specific to PmA, ‘transport’ activity was significantly enriched in Gene Ontology analysis. MapMan classification showed that, while both up- and down- regulations were observed for auxin, abscisic acid, and brassinolides signaling genes, the jasmonic acid and ethylene signaling pathway genes were all up-regulated, suggesting H_2_O_2_-enhanced JA/Et functions in PmA. To further study whether any of these genes were involved in wheat PM response, 19 H_2_O_2_-responsive putative defense related genes were assayed in wheat seedlings infected with *Blumeria graminis* f. sp. *tritici* (*Bgt*). Eight of these genes were found to be co-regulated by H_2_O_2_ and *Bgt*, among which a fatty acid desaturase gene *TaFAD* was then confirmed by virus induced gene silencing (VIGS) to be required for the PM resistance. Together, our data presents the first global picture of the wheat transcriptome under H_2_O_2_ stress and uncovers potential links between H_2_O_2_ and *Bgt* responses, hence providing important candidate genes for the PM resistance in wheat.

## Introduction

Hydrogen peroxide (H_2_O_2_) is a well known toxic molecule and is also a specific component of several biotic and abiotic signaling pathways [1]. In plants, H_2_O_2_ production causes oxidative stress during external stimuli such as chilling [2], drought [3], salinity [4], UV irradiation [5], ozone exposure [6], heavy metal [7], and wounding [8]. H_2_O_2_ is also produced upon phytohormone treatments such as abscisic acid (ABA) [9] and jasmonic acid (JA) [8], as well as during elicitor and pathogen challenges [10]–[11]. In almost all cases, H_2_O_2_ seems to be positively employed by plants to activate certain stress-responsive genes that help them cope with adverse environmental changes.

Global gene expression profiling experiments on H_2_O_2_-treated plants have revealed a large number of genes in Arabidopsis and tobacco that are mostly involved in response to oxidative stress [12]–[14]. Such treatments, however, not only affect genes involved in reactive oxygen species (ROS) detoxification, but also drive the expression of genes involved in signal transduction, transcriptional regulation and protein, carbohydrate or lipid metabolism, illustrating the complexity of the transcriptional responses to H_2_O_2_. Recently, a proteomics investigation of proteins that are differentially accumulated responding to exogenous H_2_O_2_ was performed in rice [15]. The study identified proteins that are involved in various cellular responses and metabolic processes, redox homeostasis, signal transduction, protein synthesis and degradation, photosynthesis and photorespiration, and carbohydrate/energy metabolism.

Production of ROS has been observed during pathogen infection and is also a hallmark of successful recognition of infection and activation of plant defense systems. Oxidative burst is one of the fastest defense responses activated in plants to resist pathogen and parasite attacks. It consists of the production of ROS, mainly H_2_O_2_, at the first site of pathogen invasion: the plant cell wall, and incurs a number of events before the transcription-dependent defense mechanisms are activated [16]. Subsequently, H_2_O_2_ works as a selective signal for the induction of a subset of defense genes including phosphorylation cascades [17]–[18], cyclic oxylipins of the jasmonate type [19], phytoalexins and secondary metabolites [20], as well as genes associated with programmed cell death [21] and plant hormone signaling [22]. Phyto-oxylipins are metabolites produced in plants by the oxidative transformation of unsaturated fatty acids via a series of diverse metabolic pathways and are believed to play a pivotal role in plant defenses as signal molecules and/or protective compounds [23]. Along with salicylic acid (SA), JA, and ethylene (Et) are hormones usually associated with the induction of defenses where they antagonistically interact with SA [24]. ROS are proposed to be the central component of a self-amplifying loop that regulates the interaction between SA, JA and Et to mediate the response to ozone and possibly some defense and cell death processes [25]–[26].

Wheat (*Triticum aestivum* L.) is one of the most important crops to feed the world's population. Each year, wheat powdery mildew (PM) causes serious yield loss worldwide. H_2_O_2_ has been shown to accumulate in the mesophyll cells during the early stage of the wheat-PM incompatible interaction [11], [27]–[28], suggesting that H_2_O_2_ signaling plays a role in PM defense. In Arabidopsis, the co-regulation of H_2_O_2_ signaling and defense responses was reported. For example, H_2_O_2_ regulates the coordinated action of the Arabidopsis *TGA1* (*TGACG motif-binding factor 1*) and *NPR1* (*non expressor of pathogenesis-related genes 1*) genes that are required for defense gene expression and systemic acquired disease resistance [18], [29]. Similarly, a serine/threonine kinase gene, *Stpk-V* introgressed from *Haynaldia villosa*, a wild relative of wheat, is recently reported to be induced by both *Blumeria graminis* s.sp. *tritici* (*Bgt*) and exogenous H_2_O_2_, and confers the resistance to the powdery mildew (PM) in wheat [30]. These studies demonstrate the coordination of the H_2_O_2_-induced signaling pathways and that of pathogens. The recent advance in next-generation sequencing technology provides a powerful tool for sequence-based transcriptome analysis in species with large genomes such as wheat. However, a global analysis of H_2_O_2_-responsive genes in wheat is still lacking, especially H_2_O_2_-triggered defense related genes and molecular pathways.

In the present work, we performed a transcriptome analysis of H_2_O_2_-treated wheat seedlings in the PM resistant (PmA) and susceptible (Cha and Han) lines. Using the mRNA tag sequencing approach, we identified differentially expressed genes with consistent expression patterns in all three wheat lines as well as those with specific expression patterns in PmA. We found that the basal H_2_O_2_-responsive genes in wheat were involved in various cellular responses and metabolic processes, with functions toward cell defense, signal transduction, photosynthesis, carbohydrate metabolism, lipid metabolism, redox homeostasis, and transport. In PmA, Gene Ontology analysis revealed the enrichment of ‘transport’ activities, while MapMan classification unraveled activated genes in PmA for JA and Et signaling pathway. Further, eight genes were found to be co-regulated by H_2_O_2_ treatment and *Bgt* inoculation, among which a fatty acid desaturase gene (*TaFAD*) was shown to be involved in the PM resistance. Our work is the first transcriptome-wide analysis of wheat genes responding to H_2_O_2_ treatment and provides candidate genes that may deserve further investigation for PM resistance in wheat.

## Results

### The morphological and physiological changes of wheat seedlings under H_2_O_2_ treatment

**Figure 1:**
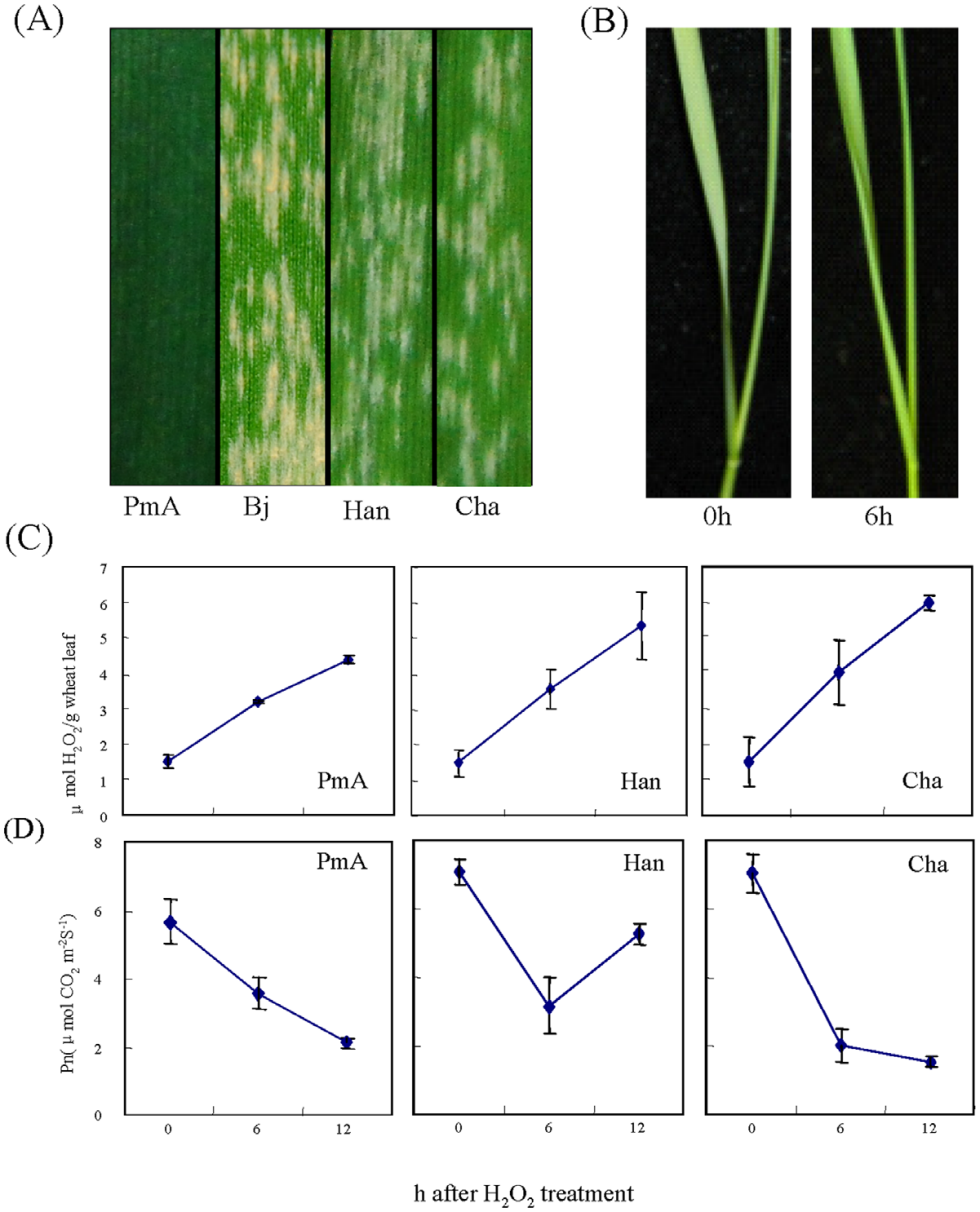
Effects of H_2_O_2_ treatment on wheat seedlings. (A) PM response of the resistance line PmAm6/Beijing837 BC5F3 (PmA) and the susceptible lines Beijing837 (Bj), Hanxuan10 (Han), and Chadianhong (Cha) at 10 day after *Bgt* inoculation. (B) Phenotypes of 9-day-old wheat seedlings after 6 h 10 mM H_2_O_2_ treatment. (C) Accumulation of endogenous H_2_O_2_ in wheat seedling leaves. (D) Suppression of photosynthesis efficiency (Pn) in wheat seedling leaves by H_2_O_2_. For (C) and (D), the data are based on three biological replicates, each with ten plants.

H_2_O_2_ can act as signaling molecules at low concentrations by diffusing into cells and be rapidly and specifically perceived by a series of target proteins before being scavenged by antioxidative defense mechanisms [15]. These H_2_O_2_ signals are transmitted to downstream signaling molecules and together modulate various metabolic and defense pathways in plants [9], [13], [31]–[32]. To study the effects of H_2_O_2_ on wheat seedlings and discover genes that are potentially involved in biotic responses, we set out to investigate the H_2_O_2_-triggered transcriptome profile changes in one PM-resistant (PmA) and two PM-susceptible lines (Cha and Han; see Materials and Methods; Figure 1A). As shown in Figure 1B, the growth of 9-day-old wheat seedlings in 10 mM H_2_O_2_ for 6 hours (h) did not cause evident changes in morphology, which was in contrast to the severely curled leaves of rice seedlings under similar conditions [15]. To confirm that the application of exogenous H_2_O_2_ indeed elevated the cytosolic H_2_O_2_ level, we determined the endogenous H_2_O_2_ concentration in the treated leaves. The results showed that the internal H_2_O_2_ level indeed increased by more than 200% over the control in all of the three wheat lines (Figure 1C). In contrast, the net photosynthetic rate (Pn) in all of the three wheat lines were decreased (Figure 1D). These measurements demonstrate that the current treatment condition was sufficient to cause physiological changes in the wheat cells. We then carried out an mRNA tag profiling analysis using RNA samples extracted from the seedling leaves. The two libraries derived from the 0 h and 6 h time points were named as follows: for PmA, PK and P6, respectively; for Han, HK and H6, respectively; and for Cha, CK and C6, respectively.

### The mRNA tag raw data processing, gene association, and differential expression analysis

An average total of 11,841,476 high quality tags (clean tags) were obtained from each of the six libraries. After removing un-mappable reads, 9,201,359 tags were shown to have at least one match (with less than 1 bp mismatch considering the existence of homoeo-alleles) in the reference tag database generated from 274,754 PlantGDB sequences (Release 163b). The unmatched tags may arise from the absence of ESTs in the current datasbase or from sequencing errors, and were not pursued further. More than 30,000 genes that were tagged by 291,026 unambiguous tags for each library were further analyzed for differential expression analysis (see Materials and methods for the data processing details, Table S1), with an average number of tags per gene as 137.9. A total of 6,157, 6,876, and 3,268 transcripts were found to be differentially expressed between 0 h and 6 h H_2_O_2_ treatment in PmA, Han, and Cha respectively (*p*<0.001 and FDR<0.001). In PmA, the number of up-regulated transcripts (4,008) was nearly 2-fold that of the down-regulated transcripts (2,148), whereas the numbers of up- and down-regulated transcripts in Han (3,780 vs 3,095) and Cha (1,780 vs 1,487) were similar (Table S2). A BlastN search between tagged ESTs and sequences presented on Affymetrix wheat GeneChips showed that only a third of these differentially expressed transcripts were represented by the latter methodology, demonstrating that the high-throughput sequencing technology-based gene expression methodology provides more comprehensive information than the traditional array-based approach.

A correlation analysis showed that the correlation coefficients (*R* ^2^) between libraries of the same wheat line were higher (>0.95) than those between different wheat lines, regardless whether the plants were treated with H_2_O_2_ or not. This result indicates significant variations in basal gene expression levels between the wheat lines of different genetic backgrounds (Table S3). This observation also cautions us to take all three of the lines into consideration when identifying genes that specifically respond to H_2_O_2_. A total of 28 differentially expressed genes between PK and P6, as measured in tag numbers, were then verified using qRT-PCR. The result showed >86% (24 out of 28 genes) consistency between the two methods (Table S4).

### Functional classification of differential expression genes with consistent expression patterns in all three wheat lines

**Figure 2:**
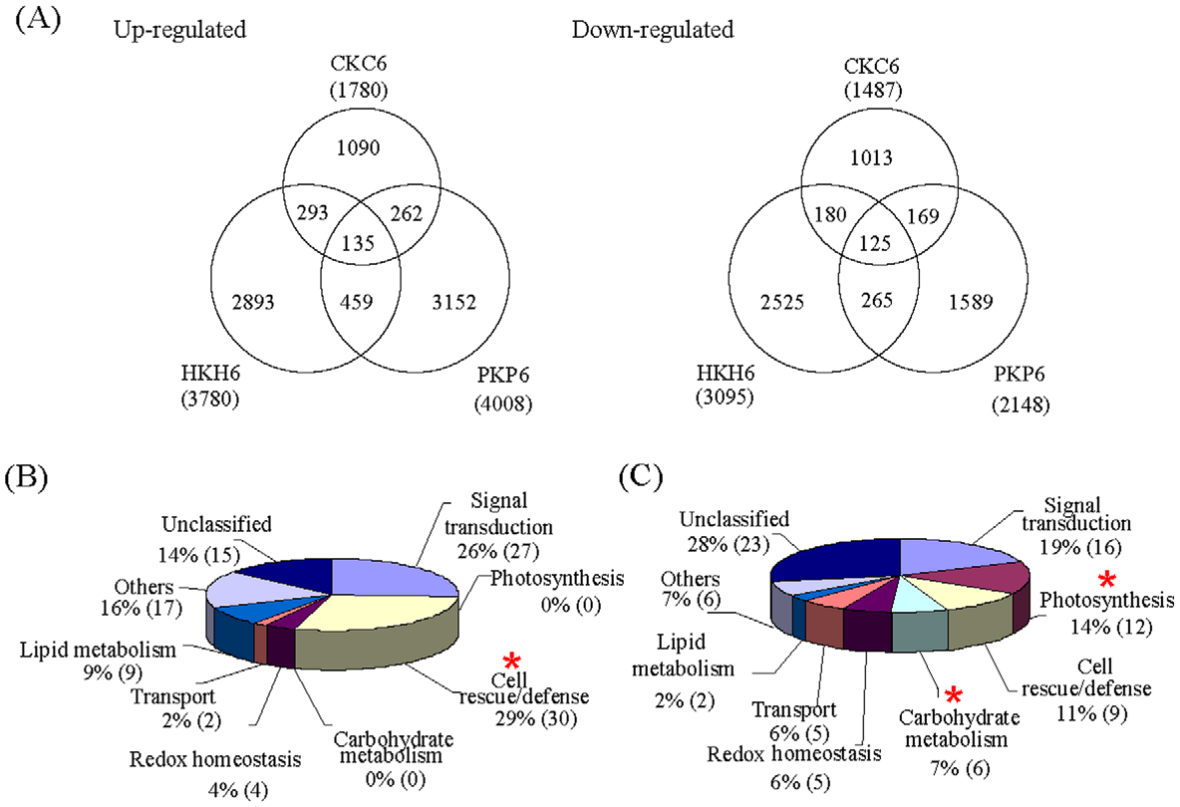
Classification of differentially expressed genes in wheat seedlings under H_2_O_2_ treatment. (A) Venn diagram analysis of the differentially expressed genes (fold change ≥2; tag number ≥12; *P* value<0.001) in PmA (PK/P6), Han (HK/H6), and Cha (CK/C6). Functional classification of 135 (104 annotated) up-regulated (B) and 125 (84 annotated) down-regulated genes (C) that are differentially expressed across all three lines. Wheat ESTs were annotated by their similarity to rice proteins. The significance between up and down regulated gene numbers was tested using Fisher's exact test. *p<0.05.

To reduce the potential variation derived from different genetic backgrounds, we firstly looked at the genes with consistent differential expression patterns in all three of the lines. A total of 260 genes fell in this category, with 135 genes up-regulated and 125 down-regulated (Figure 2A, Table S5). We then manually classified these genes using functional categories as reported [15]. The numbers of up- and down-regulated genes were significantly different (Fisher's exact test *P*<0.05) in the functional categories related to cell rescue/defense, photosynthesis, and carbohydrate metabolism (Figure 2B, 2C). For example, nearly a third (30 ESTs, or 29%) of the up-regulated genes were annotated as to be involved in cell rescue/defense, significantly more than the ones down regulated (9 ESTs, or 11%). In this category, a number of genes induced by H_2_O_2_ are clear components of defense responses, such as chitinase (Ta-1686165446), cytochrome P450 enzymes (Ta-98605, 137154, 127510), hypersensitive-induced response protein (Ta-1646165446, 265165443), late embryogenesis abundant group 1 protein (Ta-108515, 44767, 04365), wound-induced precursor (Ta-1412165445, 012207), and glycosyl hydrolases family protein (Ta-0877, 33326, 81876), whereas those down regulated are cellulase (Ta-64131), expansins (Ta-1865165444, 89717), and WAX2 (Ta-93863) genes (Table 1). In contrast, the expressions of genes for other two categories, i.e. photosynthesis and carbohydrate metabolism, were all repressed. Twelve genes (14%) were associated with photosynthesis including those for the light harvest center such as chlorophyll A-B binding proteins (Ta-0112922, 036375, 053588, 129259, 146211, 19895, 2098165442, 3264165442, 98245) and photosynthesis I and II reaction center proteins (Ta-2040165448, 132758). The consequent repression of carbohydrate metabolism genes included key sucrose synthesis genes such as beta-amylase (Ta-155349), trehalose-6-phosphate synthase (Ta-2355165445), and ribulose bisphosphate carboxylase small chain precursor (Ta-3117165446; Table S5).

**Table 1:** H_2_O_2_-responsive ESTs annotated with cell rescue/defense functions.

PlantGDB EST No.	Putative function	Fold change (log2)^a^		
		PmA	Han	Cha
**Up-regulated**				
Ta-1686165446	CHIT8 - Chitinase family precursor	1.55	1.23	1.19
Ta-28626	CSLF8 - cellulose synthase-like family F; beta1,3;1,4 glucan synthase	2.92	1.71	4.16
Ta-98605	cytochrome P450 72A1	4.02	8.21	1.85
Ta-137154	cytochrome P450	3.49	8.87	5.50
Ta-127510	cytochrome P450	2.73	2.05	1.57
Ta-760165445	dehydrin	7.50	7.75	7.17
Ta-1646165446	hypersensitive-induced response protein	2.83	3.43	2.13
Ta-265165443	hypersensitive-induced response protein	3.13	1.72	2.56
Ta-108515	late embryogenesis abundant group 1	7.56	8.64	7.29
Ta-44767	late embryogenesis abundant group 1	7.99	5.44	3.46
Ta-04365	late embryogenesis abundant group 1	1.13	1.39	1.69
Ta-85498	OsRCI2-5 - Putative low temperature and salt responsive protein	1.89	3.00	1.97
Ta-65960	pectinesterase inhibitor domain containing protein	2.97	2.23	2.52
Ta-1137165445	pectinesterase	1.38	1.44	2.02
Ta-1864165443	pleiotropic drug resistance protein	6.67	8.95	2.33
Ta-33080	thaumatin	7.50	2.49	2.73
Ta-1412165445	WIP3 - Wound-induced precursor	2.77	2.16	1.40
Ta-012207	WIP3 - Wound-induced precursor	1.60	2.11	4.19
Ta-0109885	glyoxalase family protein	2.45	2.10	1.59
Ta-36034	wound/stress protein	1.09	1.57	1.53
Ta-95374	jacalin-like lectin domain containing protein	2.08	2.47	3.40
Ta-0877	glycosyl hydrolases family 16	2.43	3.15	2.25
Ta-33326	glycosyl hydrolases family 16	4.04	2.99	1.49
Ta-81876	glycosyl hydrolases family 16	1.79	1.29	8.98
Ta-58864	glycosyl hydrolases	1.88	2.67	1.98
Ta-839165443	alpha-1,4-glucan-synthase	1.67	2.08	2.50
Ta-111845	vignain precursor	1.45	1.75	1.33
Ta-1262165444	vignain precursor	8.44	1.70	8.29
Ta-152385	lysM domain containing protein	3.21	2.17	1.54
Ta-92174	BBTI6 - Bowman-Birk type bran trypsin inhibitor precursor	6.57	7.35	3.97
**Down-regulated**				
Ta-64131	cellulase	−1.58	−2.20	−1.39
Ta-117342	cytochrome P450	−1.47	−2.49	−4.38
Ta-1865165444	expansin precursor	−9.44	−1.86	−2.11
Ta-89717	expansin precursor	−8.16	−6.44	−8.11
Ta-0115846	glycosyl hydrolases	−1.62	−2.85	−2.40
Ta-111456	late embryogenesis abundant D-34	−1.97	−3.20	−1.89
Ta-1523165444	Os1bglu5 - beta-glucosidase homologue, similar to G. max isohydroxyurate hydrolase	−1.87	−3.02	−1.70
Ta-101667	verticillium wilt disease resistance protein	−2.15	−1.61	−8.05
Ta-93863	WAX2	−1.73	−1.33	−1.99

a 6 h TPM∶0 h TPM;

Ta, PUT-163b-Triticum_aestivum; PmA, PmAm6/Beijing837 BC5F3; Han, Hanxuan10; Cha, Chadianhong.

In addition, four categories contain similar numbers of induced and repressed genes. These categories were signal transduction, lipid metabolism, redox homeostasis, and transport. Genes for signal transduction, for example, were comprised of 27 (26%) induced genes and 16 (19%) repressed ones (Table S5). These genes are of various signaling functions and encode putative calcium/calmodulin-dependent protein kinase (Ta-18711), calmodulin-related calcium sensor protein (CML) (Ta-095679), mitogen-activated protein kinase (MAPK) (Ta-60654), phosphatase 2C proteins (Ta-2846165449, 18275, 57939, 133196, 804165440, 3652165444), and AP2 transcription factors (Ta-51510, 29471), MYBs (Ta-089056, 056632, 24630), WRKY (Ta-064372), zinc-finger (Ta-9295), and bHLH (Ta-58552). Eleven genes were involved in lipid metabolism (9 up- and 2 down-regulated), including putative lipid transfer protein like (LTPs) family proteins (Ta-104587, 035988, 459165442, 66239), lipoxygenases (Ta-01383, 1776165447), fatty acid hyroxylase (Ta-45483), and acyl carrier protein (Ta-15083). There were nine genes (4 up- and 5 down-regulated) that were involved in redox homeostasis. The genes in this category encode dehydrogenases (Ta-21469, 31030), tropinone reductase 2 proteins (Ta-36863, Ta-060010), glutathione S-transferase (GST, Ta-66576), glutathione peroxidase (Ta-19785), peroxisomal membrane protein (Ta-121223), peroxidase precursor (Ta-010988), and thioredoxin (Ta-2210165449). There were seven genes that were involved in transport activity (2 up- and 5 down-regulated). The genes in this category encode MDR-like ABC transporter (Ta-2659165443), transmembrane amino acid transporter protein (Ta-151041), amino acid transporter (Ta-118377), mitochondrial carrier protein (Ta-01816), peptide transporter (Ta-149275), and trafficking particle complex subunit (Ta-95913). The genes in other category were mainly were involved in protein biosynthesis and degradation (Table S5). In addition, a group of 38 genes (15 up- and 23 down-regulated) were annotated to encode putative, hypothetical, or expressed proteins of unknown functions. Finally, we compared these H_2_O_2_-regulated genes with those differential genes from several other wheat microarray works. As shown in Table 2, a number of H_2_O_2_ regulated genes also participated in other biotic and abiotic stresses, such as heat, drought, and pathogen infections (leaf rust, yellow rust, and *Fusarium pseudograminearum*), confirming the diverse functions of H_2_O_2_ in wheat stress responses. There seem to be more common genes differentially expressed under our condition and under drought and heat conditions [33]. This may be attributed to the similar time points for sample collection in the two experiments.

**Table 2:** Co-regulation of H_2_O_2_-responsive wheat genes under other stress conditions as detected by the microarray analyses.

ArrayExpress or GEO accession	Condition	Time course	Fold change cut-off^a^	Common genes	Reference
E-MEXP-1523	Heat and drought	1 h, 3 h, 24 h	2	43	Qin et al. 2008 [33]
TA23	Drought	11 DPA	2	12	April et al. 2009 [34]
GSE6227 (TA29)	*Puccinia triticina* race MFBL (*Lr34*)	36 h	5	12	Hulbert et al. 2007 [35]
TA11	*P. striiformis f. sp. tritici* (*Yr39*)	12 h	2	2	Coram et al. 2008 [36]
TA24	Magnaporthe isolate BR32 (adapted)	24 h	2	4	Boyd 2009 [37]
GSE13346 (TA31)	*Fusarium pseudograminearum*	1 day	1.5	5	Desmond et al. 2008 [38]
GSE13660	*Blumeria graminis f. sp. Tritici*	3 weeks	1.5	1	Chain et al. 2009 [39]
TA2 (E-GEOD-12508)	Seedling root vs seedling leaf	–	8	5	Schreiber et al. 2009 [40]

a As used by the authors or selected to make a dataset of a reasonable size.

### Gene Ontology analysis suggests H_2_O_2_-enhanced transport activities in PmA

In barley and wheat, H_2_O_2_ can accumulate in the mesophyll cells during the early stage of the host-PM incompatible interaction, where it acts as an important signaling molecule to activate defense systems [11], [27]–[28]. Since PmA is resistant to *Bgt* isolate E09 while Han and Cha are susceptible, we studied in PmA H_2_O_2_ responding genes that were “subtracted” with their expression patterns in Han and Cha. We defined three classes of PmA-specific (PmA-H_2_O_2_) genes: class I comprises genes whose expression was induced in PmA (U) but suppressed (D) or remained unchanged (N) in both Han and Cha (UDD/UNN); class II includes genes that were suppressed in PmA (D) but induced or unchanged in Han and Cha (DUU/DNN); class III contains genes whose expression was not affected in PmA but changed in Han and Cha (NUU/NDD) (Table S6, S7). A total of 2,982 genes fell in these categories. Among them, nearly 60% of the genes (1,763) belong to class I genes (fold change ≥2, *p*<0.001, and FDR<0.001), while classes II and III genes comprise 28% (846) and 12% (373) respectively.

Gene Ontology (GO) analysis showed that class I genes were mostly enriched in functions associated with the biological process (BP) terms “localization”, “response to stimulus”, and “metabolic process”, the molecular function (MF) terms “transporter activity”, “catalytic activity” and “binding”, and the cellular component (CC) terms “plasma membrane”, “vacuole” and “membrane-bounded organelle” (Table 3). A total of 112 genes were associated with “localization” including 43 transporters, 18 protein targeting proteins, and 11 cell vesicle transporters (data not shown), among which a total of 21 genes were annotated as related to the vesicle-mediated transport (Table S8). Genes with “transporter activity” were 17.5 fold more over-represented than in the Arabidopsis ATH1 Genome Array ATH1 (Table 3) that contains more than 22,500 probe sets representing approximately 24,000 genes. Transporter genes are known to be associated with defense responses by participating in the formation of multi-vesicular bodies and cell wall-associated paramural bodies that have been shown to be involved in secretion of building blocks for cell wall appositions [41]. These vesicle bodies not only arrest fungal penetration but also can cause hypersensitive cell death through blocking plasmodesmata. However, whether H_2_O_2_ enhanced membrane transport, as observed here in wheat, plays a role in pathogen defense or not may need further experimental investigation.

**Table 3:** Enriched GO terms among H_2_O_2_-responsive genes in the PM resistant line PmA.

GO terms	Percent of total transcripts		H_2_O_2_ over representation	P value	FDR
	This Study	ATH1			
**UNN/UDD (Class I)**					
***Biological Process***					
localization	0.12	0.05	2.4	6.90E-15	3.40E-12
response to stimulus	0.18	0.11	1.6	9.40E-10	1.10E-07
metabolic process	0.41	0.28	1.5	2.70E-10	3.50E-08
***Molecular Function***					
transporter activity	0.7	0.04	17.5	1.80E-06	2.70E-04
catalytic activity	0.4	0.26	1.5	2.10E-13	1.90E-10
binding	0.4	0.3	1.3	1.60E-06	2.70E-04
***Cell Component***					
plasma membrane	0.2	0.04	5	3.80E-40	2.10E-37
vacuole	0.05	0.01	5	2.30E-19	9.80E-18
membrane-bounded organelle	0.4	0.2	2	8.20E-27	1.10E-24
**DNN/DUU (Class II)**					
***Biological Process***					
localization	0.1	0.05	2	1.20E-04	3.90E-03
cellular process	0.5	0.3	1.7	4.30E-06	3.30E-04
metabolic process	0.45	0.3	1.5	2.60E-07	3.30E-05
***Molecular Function***					
electron carrier activity	0.03	0.007	4.3	8.80E-04	3.10E-02
catalytic activity	0.4	0.3	1.3	2.50E-09	9.90E-07
binding	0.4	0.3	1.3	1.40E-03	3.50E-02
***Cell Component***					
chloroplast stroma	0.06	0.006	10	2.80E-14	1.10E-12
chloroplast thylakoid membrane	0.05	0.006	8.3	5.20E-12	7.50E-11
plasma membrane	0.1	0.04	2.5	5.90E-09	5.30E-08
**NUU/NDD (Class III)**					
***Biological Process***					
cellular component biogenesis	0.06	0.02	3	3.00E-04	1.70E-02
response to stimulus	0.2	0.1	2	2.80E-05	3.80E-03
metabolic process	0.5	0.3	1.7	1.50E-05	2.60E-03
***Molecular Function***					
catalytic activity	0.5	0.3	1.7	5.30E-07	1.20E-04
***Cell Component***					
chloroplast stroma	0.06	0.006	10	1.40E-07	3.80E-06
organelle membrane	0.06	0.02	3	6.10E-03	3.70E-02
plasma membrane	0.1	0.04	2.5	8.10E-06	8.80E-05

GO analysis showed that class II genes were also enriched with “localization” and “metabolic process” (BP), “catalytic activity” and “binding” (MF), and “plasma membrane” (CC). The difference between the class II and the class I genes was the enrichment of the GO terms “chloroplast stroma” (CC, 10 fold enrichment) and “thylakoid membrane” (CC, 8.3 fold enrichment) in class II genes, suggesting significant effects on chloroplast functions by H_2_O_2_. The composition of the class III genes (up- or down-regulated only in both Han and Cha) was enriched with the BP term “cellular component biogenesis” and the CC term “chloroplast stroma” (10 fold enrichment). In contrast, the BP “localization” was not enriched among the genes of this category, demonstrating a difference in membrane transport activities between the PM susceptible and resistant lines. Therefore, these data point to possible roles of H_2_O_2_ in pathogen defense, with enhanced vesicle transportation as one of the possible approaches.

### MapMan analysis showed H_2_O_2_-enhanced JA/Et signaling pathway in PmA

**Figure 3:**
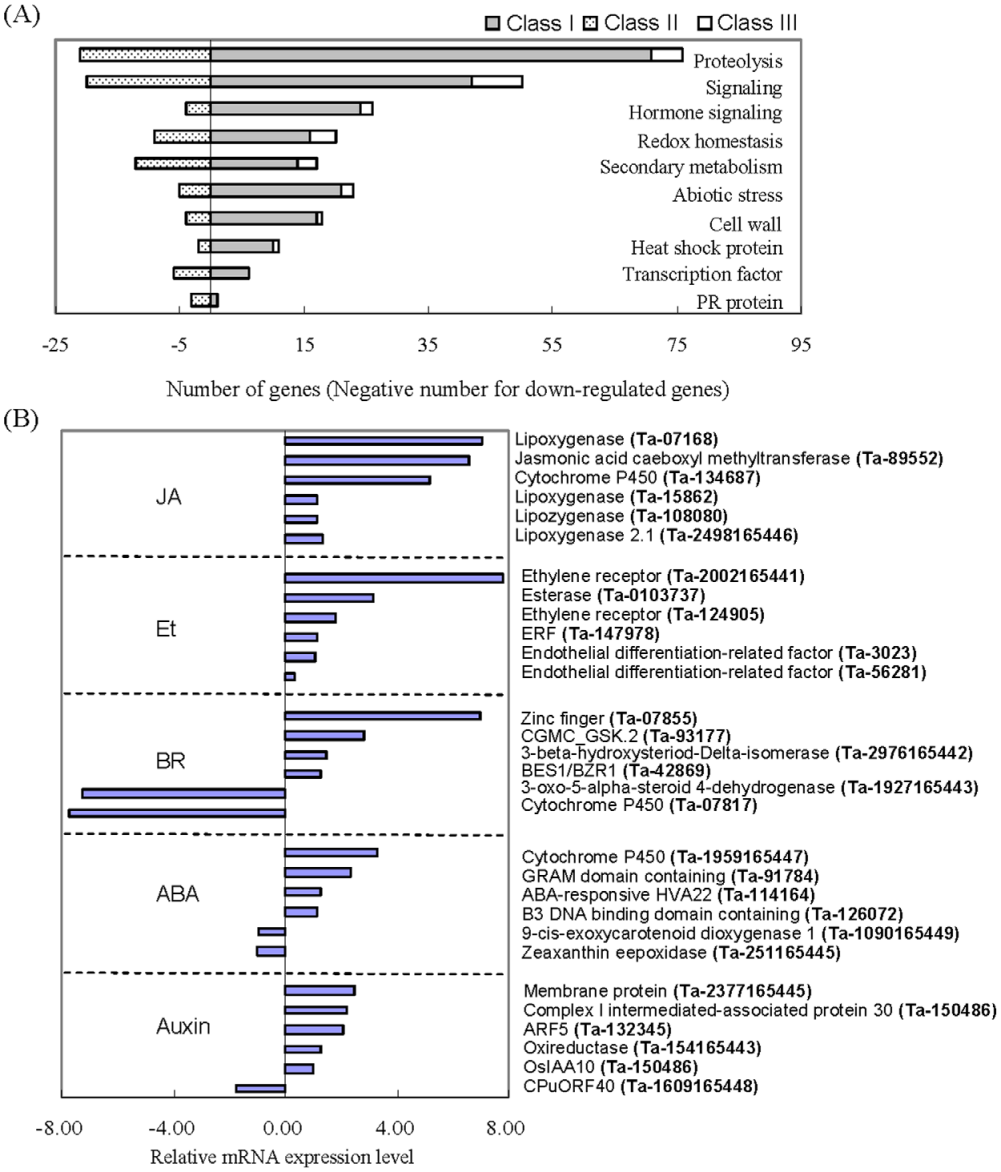
Functional categories of MapMan biotic stress overview of differentially expressed genes specific to the PM resistant line PmA. (A) An overview of genes involved in biotic stress. Genes of classes I, II, and III are as defined in the text. Numbers of down regulated genes are represented by negative numbers. Genes in each functional category were listed in Table S10. (B) List of genes involved in hormone signaling and their relative expression levels between 0 h and 6 h H_2_O_2_ treatment. The expression levels are represented as the log_2_ value of the mRNA tag number ratios between the two libraries.

To study the possible roles of H_2_O_2_ responding genes in biotic response, we overlaid classes I, II, and III genes to the Arabidopsis GeneChip template in the MapMan program [42] and analyzed them in the biotic stress overview (Figure S1). Figure 3A summarizes 10 major categories of genes that were associated with biotic response according to the MapMan classification. Among these, the most abundant genes (102 ESTs) were involved in protein degradation of various functions such as subtilases, cysteine protease, aspartate protease, serine protease, metalloprotease, AAA type, ubiquitin E1, ubiquitin E2 and ubiquitin E3 (Table S9, S10). Ubiquitination-associated proteins have been shown to play important roles in plant-microbe interactions [43]. The second group of genes (75 ESTs) was associated with signaling including more than 30 receptor kinases, calcium/calmodulin dependent kinases, and ras-related proteins (Figure 3A; Table S10). Protein kinases are well known to play a central role in the pathogen recognition and subsequent activation of plant defense [44]. The biotic stress overview also displays genes involved in redox homeostasis (31 ESTs), secondary metabolism (31 ESTs), cell wall (23 ESTs), heat shock proteins (13 ESTs), pathogenesis-related proteins (PRs; 4 ESTs), and 31 ESTs that may be overlapping with additional abiotic responses.

A total of 30 genes were involved in hormone signaling according to their annotation. As shown in Figure 3B, except for salicylic acid (SA), putative signaling genes for auxin, abscisic acid (ABA), JA, Ethylene (Et), and brassinolides (BR) were all affected by H_2_O_2_ among PmA-H_2_O_2_ genes. For ABA, Auxin, and BR, some genes were up regulated whereas others were down regulated. In contrast, all JA and Et signaling genes were up regulated, indicating that H_2_O_2_ is able to enhance JA/Et signaling in PmA (the two hormones often work together) [45]–[47]. The 12 putative JA/Et signaling genes encode lipoxygenases (LOXs, Ta-07168, 15852, 2498165446), lipozygenase (Ta-108080), allene oxide synthase 2 (AOS2, Ta-134687), jasmonic acid carboxyl methyltransferase (JMT, Ta-89552), ethylene receptors (ETRs, Ta-2002165441, 124905), esterase (Ta-0103737), ethylene response factor (ERF, Ta-147978), and endothelial differentiation-related factors (Ta-3023, 56281; Figure 3B). Six of the above 12 JA/Et genes (Ta-2498165446, 108080, 134687, 89552, 2002165441, 147978) were selected for qRT-PCR confirmation and, as expected, were all induced by H_2_O_2_ (Table S11). Besides, two additional JA biogenesis-related genes Ta-80972 and Ta-04807 were also confirmed by qRT-PCR to be up regulated by H_2_O_2_ (Table S11). Ta-80972 encodes a cytochrome P450 that is highly similar to a rice allene oxide synthase (AOS), a key enzyme in the oxylipin pathway leading to AOS-derived jasmonates, while Ta-048072 encodes the enzyme 12-oxophytodienoate (OPDA) reductase that is involved in JA biosynthesis by catalyzing the reduction of 10, 11-double bonds of OPDA to yield 3-oxo-2-(2′-pentenyl)-cyclopentane-1-octanoic acid (OPC-8:0). These results suggest that it is not only the signaling, but also the biogenesis of JA that may have been enhanced in the H_2_O_2_-treated wheat.

### Co-regulation of H_2_O_2_ responsive genes by *Bgt* inoculation in PmA

**Figure 4:**
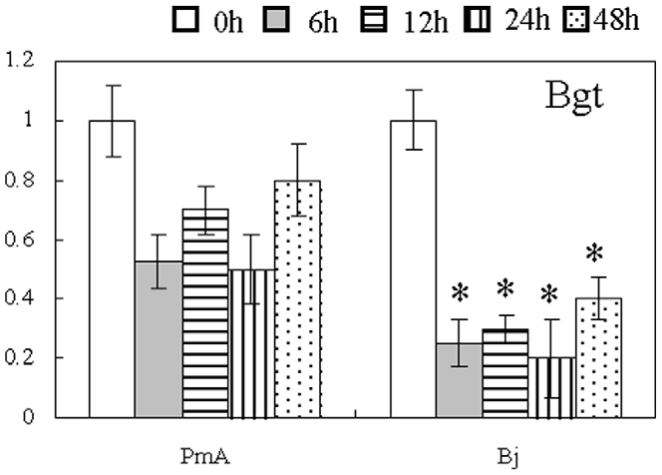
Response of *TaFAD* to the *Bgt* treatment. Expression patterns of *TaFAD* in the PM resistant (PmA) and the susceptible (Bj) near isogenic lines under *Bgt* inoculation as detected by qRT-PCR. ** *p*<0.001; * *p*<0.05.

Several studies demonstrate that H_2_O_2_-induced genes can also be regulated by pathogen infections, including powdery mildew [18], [29]–[30]. To identify H_2_O_2_ regulated genes that may also respond to wheat *Bgt* infection, 19 PmA-H_2_O_2_ ESTs that were annotated as related to defense response were selected for expression study using qRT-PCR assay between PmA and its susceptible isogenic line Beijing837 (Bj; Table 4). Eight of these genes were found to have similar expression patterns between H_2_O_2_ treatment and *Bgt* inoculation. Among them, five were class I genes including Ta-048072, 147978, 019566, 92123, and 02061 that encode 12-oxophytodienoate reductase (JA pathway), ethylene-responsive transcription factor (Et pathway), calcium/calmodulin dependent kinases (signaling), heavy metal-associated domain containing protein (cell rescue/defense), and stripe rust resistance Yr10-associated (cell rescue/defense) respectively. In the mean time, two class II genes Ta-138137 and Ta-2126165445 that respectively encode a helix-loop-helix DNA-binding domain containing protein (signaling) and an ABC transporter family protein (transport) were co-suppressed by both treatments. These H_2_O_2_ and *Bgt* co-regulated genes may be involved in the PM responses. The last EST Ta-0109540 represents a gene encoding a fatty acid desaturase (named *TaFAD*) and is a class III gene that was significantly repressed by H_2_O_2_ in the two PM susceptible lines Han and Cha (Table 4). In PmA, the fold change of the TaFAD expression under 0 and 6 h H_2_O_2_ treatments was calculated as close to 0.5, but was not significant under our statistic threshold (*p*<0.001;). qRT-PCR assay showed that under *Bgt* inoculation, *TaFAD* was significantly (*p*<0.05) repressed by *Bgt* infection in the PM susceptible isogenic line Bj (6 h∶0 h ratio 0.25±0.08) when the change in PmA was marginal (6 h∶0 h ratio 0.53±0.09). A more detailed analysis over a 48 h *Bgt* inoculation course showed that *TaFAD* was indeed significantly repressed by *Bgt* infection in Bj (*p*<0.05), whereas in PmA the modest down regulation was statistically insignificant (Figure 4). These data demonstrate that the capability to maintain a constant expression level of *TaFAD* might be essential for PM resistant wheat lines.

**Table 4:** Expression patterns of selected H_2_O_2_-responsive genes under *Bgt* inoculation in the near isogenic lines PmA and Bj.

PlantGDB EST	Annotation	Class	H_2_O_2_ treatment				*Bgt* inoculation	
			PmA^a^	Han^a^	Cha^a^	PmA^b^	PmA^b^	Bj^b^
Ta-048072^c^	12-oxophytodienoate reductase	I	**3.72**	1.84	1.33	**3.15±1.07** d	**2.95±0.44**	14±2.21
Ta-18253	SGS domain containing	I	12.21	1.23	1.06	5.2±0.13	0.08±0.02	0.13±0.01
Ta-147978	Ethylene-responsive transcription factor	I	**2.19**	0.85	0.91	**2.11±0.07**	**2.5±0.21**	2.35±0.49
Ta-3577165441	Acyl-CoA thioesterase 2	I	2.03	0.44	0.34	2.3±0.18	0.34±0.06	0.56±0.21
Ta-1259165445	Serine/threonine- kinase PRP4	I	2.97	0.40	0.01	2.83±0.15	1.57±0.59	1.23±1.01
Ta-129760	Ethylene-responsive element-binding	I	3.01	0.37	0.26	5.33±1.92	1.08±0.71	0.92±0.27
Ta-019566	CAMK_KIN1/SNF1/Nim1_like.38 - CAMK	I	**3.6**	0.33	0.15	**2.37±0.31**	**15.8±4.21**	21.9±3.72
Ta-1676165442	Solute carrier family 35 member B1	I	2.31	0.32	0.35	3.23±1.15	0.57±0.23	0.67±0.32
Ta-92123	Heavy metal-associated domain containing	I	**2.73**	0.31	0.19	**2.78±0.21**	**2.53±0.12**	2.36±1.09
Ta-99839	Peptide transporter PTR2	I	3.71	0.21	0.00	2.01±0.27	0.59±0.22	0.45±0.12
Ta-02061	Stripe rust resistance Yr10	I	**5.54**	0.01	0.00	**3.55±0.43**	**4.47±1.03**	4.92±1.23
Ta-121165441	F-box/LRR-repeat protein 14	II	0.01	163.1	263.2	0.17±0.01	2.13±1.11	1.85±0.24
Ta-4496	Hcr2-5D	II	0.01	4.41	6.88	0.23±0.06	1.62±0.52	1±0.15
Ta-099263	Thioesterase family protein	II	0.12	5.35	4.89	0.32±0.04	1.96±1.12	0.71±0.11
Ta-1039165449	OsIAA12 - Auxin-responsive Aux/IAA gene family member	II	0.24	4.44	156.5	0.12±0.09	1.26±0.44	0.9±0.33
Ta-138137	Helix-loop-helix DNA-binding domain containing protein	II	**0.37**	19.29	324.0	**0.29±0.05**	**0.02±0.02**	0.06±0.04
Ta-2126165445	ABC transporter family protein	II	**0.37**	2.13	2.79	**0.24±0.13**	**0.4±0.21**	0.28±0.22
Ta-0109540	Fatty acid desaturase	III	0.5^e^	**0.23**	**0.07**	1.25±0.22^e^	0.53±0.09^e^	**0.25±0.08**
Ta-3709	Homeobox and START domains containing	III	1.30	0.01	0.01	1.52±0.33	2.05±0.05	1.94±5.63

a Fold change detected by mRNA tag profiling (6 h TPM∶0 h TPM);

b Fold change determined by real time PCR (6 h∶0 h);

c Ta, PUT-163b-Triticum_aestivum;

d Standard error shown here indicates three biological repeats; Bolded numbers represent co-activation or co-suppression;

e indicates that the change is statistically insignificant.

**Figure 5:**
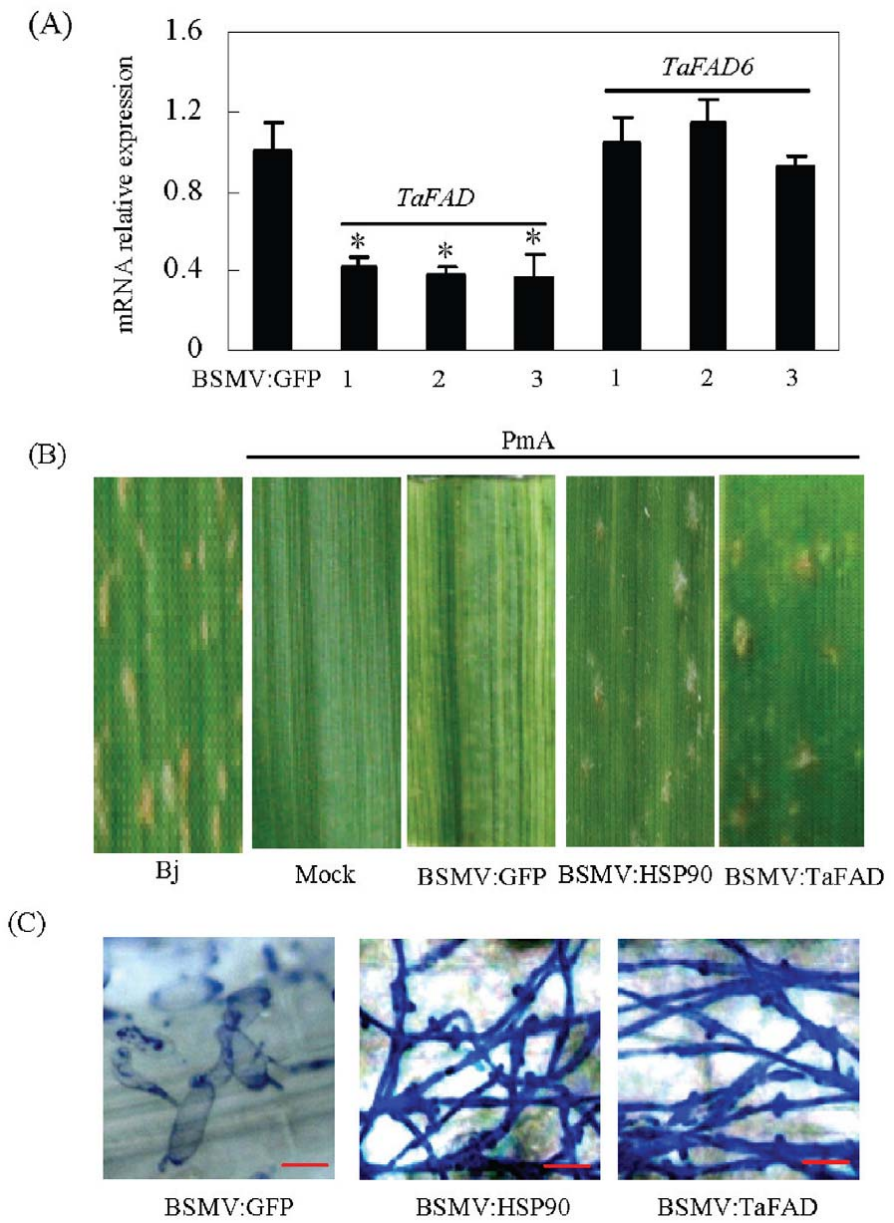
Virus Induced Gene Silencing (VIGS) assay of *TaFAD*. (A) Significant down regulation of *TaFAD* (left) in PmA plants by BSMV∶TaFAD inoculation. The expression level of another fatty acid desaturase gene *TaFAD6* (right) was not affected. (B) Susceptibility of PmA leaves to *Bgt* inoculation when *TaFAD* was “silenced” by VIGS. Bejing837 (Bj) was used as a control for successful *Bgt* infection; Mock, GKP buffer; *BSMV∶GFP*, empty vector-like control; *BSMV∶HSP90*, positive control for VIGS; At least fifteen VIGS plants were tested for each vector. (C) Microscopic observation of leaves from VIGS plants after 5 days of *Bgt* inoculation showing elongated secondary hyphae in VIGS plants. Short bars represent 10 µm. Student's *t* test *p* value *<0.05.

In Arabidopsis and rice, FADs have been shown to be involved in disease resistance by modulating JA and SA signaling [48]–[49]. To study whether *TaFAD* may play a role in wheat PM resistance, we silenced *TaFAD* in PmA using virus-induced gene silencing (VIGS) technique. A 231-bp fragment comprising the 3′ end of the ORF and part of the 3′ UTR sequence was used to increase the specificity, [50]–[51]. The results showed that down-regulation of *TaFAD* caused the compatibility between *Bgt* and PmA (Figure 5). The reduction of *TaFAD* expression resulted in the growth of *Bgt* spores on the leaves of the BSMV∶TaFAD plants and enhanced PM penetration efficiency (PE, 28% vs. 0%; Table S12). To ensure that no additional genes were affected by “off target” silencing, we also tested the expression level of *TaFAD6*, the next closest gene of *TaFAD* in wheat, and indeed no co-suppression was observed (Figure 5A). These data thus suggest that the constant expression of *TaFAD* is indeed required for the PM resistance in PmA. Since FADs have been shown to be involved in disease resistance by modulating JA and SA signaling, we then investigated wheat genes homologous to functionally characterized rice marker genes in JA and SA signaling pathways in these VIGS plants. The SA-signaling gene used was the acidic pathogenesis-related protein gene *PR1a* (Table S13), whereas the JA-signaling genes encode a lipoxygenase (*LOX*, Ta-2498165446), an allene oxide synthase 2 (*AOS2*, Ta-134687), and a pathogenesis-related protein PR1b which acts in both the JA- and SA-dependent pathways [52]–[53]. As shown in Table S13, the JA pathway related genes, Ta-134687 (*AOS2*), Ta249865446 (*LOX*), and Ta-141027 (*PR1b*) were significantly down-regulated in *BSMV∶TaFAD* plants, whereas the expression level of *PR1a* remained unchanged. Therefore, we deduce that the loss of PM resistance in *BSMV∶TaFAD* plants was likely caused by the failure to maintain proper JA signaling. *BSMV∶HSP90* was used as a positive control for successful silencing of the disease resistance system because *HSP90* is essential for the plant hypersensitive reaction (HR) to diverse pathogens as shown by previous studies [50]–[51], [54]–[56]. In *BSMV∶TaHSP90* VIGS plants, *AOS2* and *LOX* were significantly up-regulated, whereas *PR1a* were down-regulated (Table S13), suggesting different regulatory modes of *TaFAD* and *TaHSP90*. Overall, these observations suggest the potential modulation of *TaFAD* on JA signaling during wheat powdery mildew infection, which deserves further investigation.

## Discussion

In plant cells, H_2_O_2_ plays a dual role as a toxic by-product of normal cell metabolism and as a regulatory molecule in stress perception and signal transduction [15]. A complex interplay between H_2_O_2_ and other signaling molecules is known to exist in plant cells, which explains the versatility of H_2_O_2_ functions in different scenarios. However, the knowledge about the transcriptome changes by exogenous H_2_O_2_ treatment in wheat is limited, especially the knowledge about the H_2_O_2_-trggered defense related genes and signaling pathways. In this work, we performed a mRNA tag analysis of the wheat seedling transcriptome after 6 h 10 mM H_2_O_2_ treatment using the next-generation sequencing technology. We found that H_2_O_2_ caused differential expression of genes associated with important biological processes such as cell rescue/defense responses, photosynthesis, and carbohydrate metabolism. Further analysis in the PM resistant wheat line provided additional clues about the H_2_O_2_-triggered molecular pathways and the possible links of H_2_O_2_ signaling and *Bgt* defense.

### The effects of exogenous H_2_O_2_ on gene expression profiles in wheat

In rice, the exposure of seedlings to exogenous H_2_O_2_ caused leaves to curl severely [15]. By contrast, the wheat seedlings did not exhibit evident morphological changes after growth in H_2_O_2_ solution for 6 hours. Consistent with this, we found only three percent (9 out of 260) differentially expressed genes related to redox detoxification in the transcripts with identical expression patterns in all three wheat lines, suggesting that dramatic oxidative stress had not taken place under the current experimental condition. Despite this, exposure of wheat seedling roots to H_2_O_2_ stress did result in increased endogenous H_2_O_2_ accumulation with impaired photosynthesis in wheat leaves. A total of 12 genes were associated with the photosynthetic process and were all down-regulated, together with 6 carbohydrate metabolism genes, indicating that H_2_O_2_ can effectively repress photosynthesis and related carbohydrate metabolism in wheat.

Several studies show that exogenous H_2_O_2_ can initiate signal transduction processes in treated plants that render them to acquire tolerance to various abiotic and biotic stresses. In Arabidopsis, for example, genes responding to exogenous H_2_O_2_ are also involved in wilting, UV irradiation, and elicitor challenge response, indicating that H_2_O_2_ can mediate cross-tolerance toward other stresses [12]. Extra H_2_O_2_ caused by high light in a catalase-deficient Arabidopsis mutant can regulate the transcription of two clusters of genes that encode heat shock proteins (HSPs) known to be involved in stress responses [14]. Similarly, treatment of winter wheat with low concentrations of H_2_O_2_ and catalase inhibitor may enhance its tolerance to low temperature [57]. We found significant activation of putative cell rescue/defense genes than repression in the same functional category, indicating that, as in a number of other studies, H_2_O_2_ can stimulate defense responsive genes in wheat. Further comparative analysis showed that H_2_O_2_-responsive genes also participated in the response to other biotic and abiotic stresses, such as heat, drought and pathogen infections, indicating that at least some of these genes were indeed involved in defense responses.

### The characteristics of H_2_O_2_ responses in the powdery mildew resistant wheat

The accumulation of H_2_O_2_ in the mesophyll cells has been observed in barley and wheat during the early stage of the host-PM incompatible interaction, indicating that H_2_O_2_ works as an important signaling molecule in defense system activation [11], [27]–[28]. Two events were observed during this process: the accumulation of vesicles and vesicle-like materials at the cells in contact with microorganisms [41], [58] and the subsequent hypersensitive response (HR) in the epidermis cells directly nearby the penetration sites [11], [27]. We studied the differential gene responses in the PM resistant line PmA by GO enrichment analysis and found the “localization” was the most enriched BP term, together with the most enrichment GO MF term “transporter activity” and GO CC term “Plasma membrane” and “vacuole”. The enrichment of these GO terms suggest that H_2_O_2_ enhanced the membrane trafficking, supporting the hypothesis that trafficking of membrane-bound solutes, such as H_2_O_2,_ is essential not only for signaling but also for accommodating the cellular volume changes associated defense response [59].

It is nowadays impossible to discuss ROS and redox signaling in plants without considering plant hormones and related signal molecules because these compounds act together with redox-modulated signaling pathways to process and transmit environmental cues into appropriate responses. Compounds strongly interacting with redox processes include classical hormones such as auxin, ethylene, and ABA, as well as defense-related signals such as SA and JA [60]–[65]. JA is synthesized from α-linolenic acid in chloroplast membranes. The three chloroplast-located enzymes 13-lipoxygenase (13-LOX), 13-allene oxide synthase (13-AOS), and the AOC catalyze the first half of JA biosynthesis up to the intermediate product cis-(+)-12-oxophytodienoic acid (OPDA). The Arabidopsis AOS promoter is activated by a variety of signals including jasmonic acid, wounding, OPDA, and SA, indicating that regulation of the AOS gene might exert a major control on JA signaling [66]. As for OPDA, it alone is sufficient to induce defense responses. The methylation of jasmonic acid to MeJA is catalyzed by an S-adenosyl-L-methionine∶jasmonic acid carboxyl methyltransferase (JMT) in Arabidopsis. The expression of JMT itself is sufficient to induce some JA-dependent responses. In our study, we simultaneously detected the up-regulation of JA signaling and biogenesis genes including Ta-80972 (AOS), Ta-048072 (OPDA), Ta-07168, 15852, 2498165446 (LOX), and Ta-89552 (JMT). We also detected the activation of an ethylene receptor (ETR, Ta-2002165441) and an ethylene-responsive transcription factor (ERF, Ta-147978) by H_2_O_2_ treatment in PmA. It has been shown that JA, alone or often in combination with Et, mainly works in defense to insect wounding and necrotrophic pathogen attack [45]–[47]. Our results suggest that H_2_O_2_ may play a role in biotic stress by enhancing JA and/or Et signaling pathways. Whether these responsive genes also play any function in response to the infection of the biotrophic pathogens such as wheat PM is worth further investigation.

### The potential links between H_2_O_2_ signaling and *Bgt* defense

The recent identification of a serine/threonine kinase gene, *Stpk-V* as a PM resistant gene in wheat, provides further evidence that H_2_O_2_ and *Bgt* co-regulated genes can be involved in disease resistance [30]. This gene however was not detected in our work, probably because it was an introgressed gene from *Haynaldia villosa* and did not exist in the wheat line used here. In our study, eight genes exhibited similar expression patterns under H_2_O_2_ treatment and *Bgt* inoculation. These representative genes belong to various functional categories such as cell rescue/defense, signaling, JA/Et signaling pathways, transport, and lipid metabolism, and may participate in the PM defense. We show that the H_2_O_2_ responsive fatty acid desaturase gene *TaFAD* is indeed involved in the PM resistance, indicating that, as in other plants, fatty acids play important roles in wheat pathogen defense. In Arabidopsis, oleic acid (18∶1) has been implicated to participate in SA and JA-mediated defense pathways [48], [67]–[70]. It has been shown that suppressing the gene for stearoyl acyl carrier protein fatty acid desaturase (SACPD) enhances the resistance of Arabidopsis (*SSI2*), soybean, and rice to multiple pathogens [48]–[49], [70]. In addition, another fatty acid desaturase gene *FAD7* is required for the accumulation of the systemic acquired resistance-inducing activity [71], suggesting that different FAD family members may play distinct roles in plant defense. Therefore, the role of *TaFAD* in wheat PM resistance is not accidental since this gene is significantly repressed by both H_2_O_2_ and *Bgt* in the PM susceptible lines Han, Cha and Bj while maintained a relative stable expression level in the PM resistant line PmA. The observation that silencing of *TaFAD* causes the loss of PM resistance in PmA and the significant down-regulation of the JA signaling pathway supports the idea that the maintenance of *TaFAD* function is crucial for the defense response, probably due to its potential regulatory role in modulating JA signaling. Whether or how H_2_O_2_ regulates the *TaFAD* expression in PmA during *Bgt* infection needs further investigation.

### An overview of the transcriptome changes caused by exogenous H_2_O_2_ in wheat

**Figure 6:**
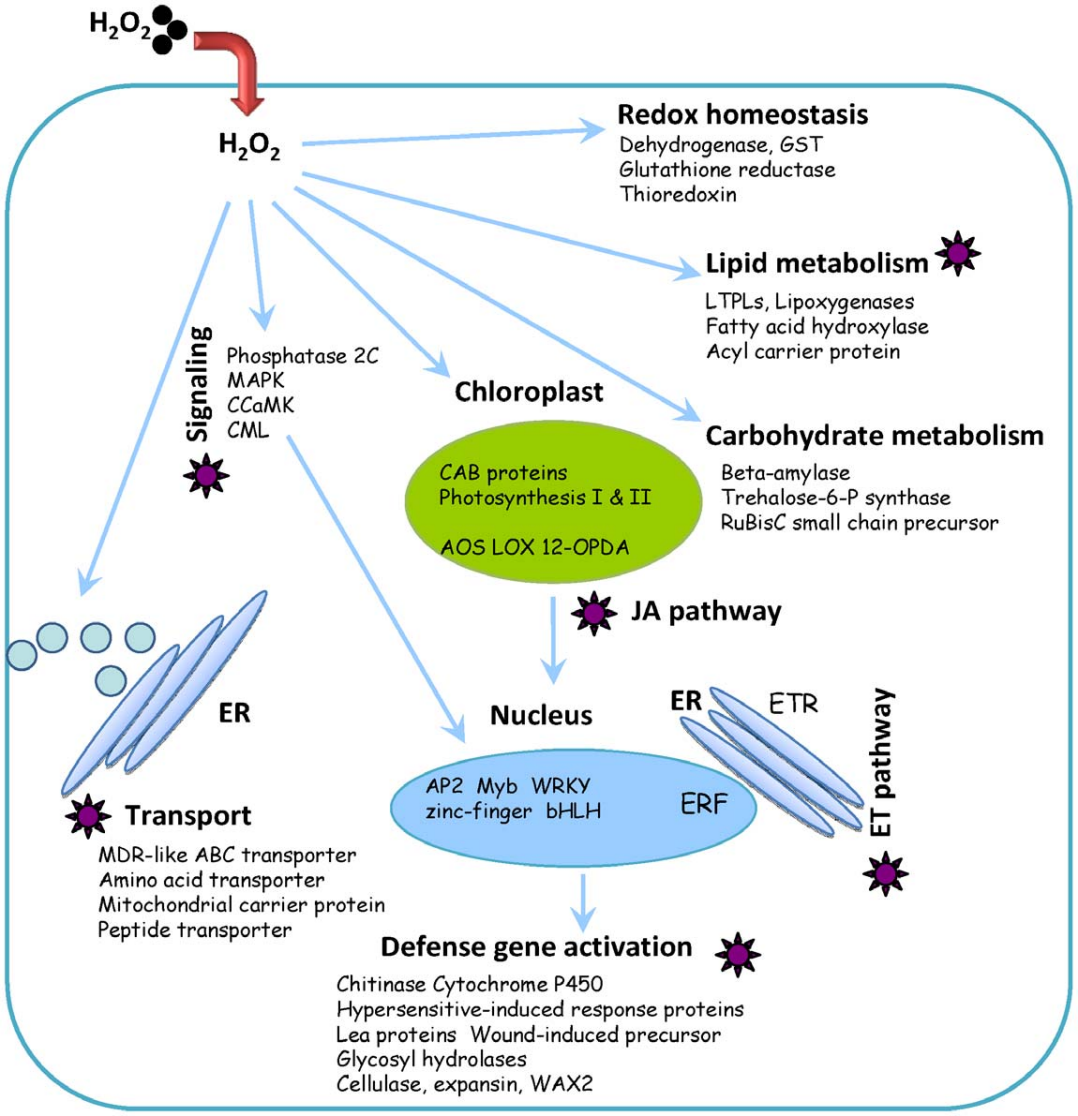
An overview of the molecular pathways and cellular processes in response to the H_2_O_2_ treatment in the bread wheat. Displayed are common molecular pathways among all three wheat lines studied that may represent basal cellular response under exogenous H_2_O_2_ treatment, except for the JA/Et pathway which is specific for PmA. Pathways labeled by stars are postulated to be also involved in biotic responses according to their enrichment in PmA. MAPK, mitogen-activated protein kinase; ROS, reactive oxygen species; AOS, allene oxide synthase; LOX, lipoxygenase; 12-OPDA, 12-oxophytodienoate; ETR ethylene receptor; ERF, ethylene-responsive transcription factor; JA, jasmonic acid; ET, ethylene; CAB proteins, chlorophyll A–B binding proteins; Lea proteins, late embryogenesis abundant group 1 proteins; LTPLs, lipid transfer protein like family proteins; ER, endoplasmic reticulum.

Several issues that are intrinsic to the hexaploid nature of the bread wheat need to be taken into consideration. For example, the mRNA tag numbers should represent the collective levels of the three homoeologous alleles if they were all expressed. Although consensus primers can be applied during qRT-PCR when sequences for all three alleles are available, allele-specific studies will result in more precise conclusions. Despite this, our analysis for the first time provides a global insight about the transcriptomic response to exogenous H_2_O_2_ treatment in wheat seedlings. Combining our observations and the previous studies, we present in Figure 6 several major cellular and metabolic processes in response to H_2_O_2_ treatment in wheat. First, the application of exogenous H_2_O_2_ may increase the cytosolic H_2_O_2_ level causing the disruption of the redox homeostasis that can be relayed by MAPKs and type-2C protein phosphatase (PP2C) or perceived by the chloroplasts where both photosynthesis and carbohydrate metabolism are consequently suppressed. Second, the MAPK cascade and/or the retrograde signals from the chloroplast are then transmitted into the nucleus where the activation of transcription factors may initiate defense related genes. Third, the enhanced JA and Et signaling, accompanied with largely up regulated lipid metabolism (which may provide needed JA precursors), may positively regulate genes for defense response where H_2_O_2_-enhanced trafficking of membrane-bound solutes may also play an important role. Finally, we propose that possible links of H_2_O_2_ signaling and *Bgt* defense may exist in the processes of signaling, transport, JA/Et pathway, and lipid metabolism (indicated by stars). Genes involved in these aspects of H_2_O_2_ response may deserve further study for their roles in the PM resistance in wheat.

## Materials and Methods

### Plant Growth and Treatments

Am6 is a synthetic amphiploid derived from a cross between *Triticum durum* (AABB) accession DR147 and *Aegilops tauschii* (DD) accession Ae39 [72]. The line used for this study (PmA) is a BC5F3 progeny between Am6 and the cultivar Beijing837 with a novel powdery mildew resistant gene. PmA is resistant to the popular *Blumeria graminis* f. sp. *tritici* (*Bgt*) race No. 15 in the Beijing area, whose virulence type is E09, while Beijing837 is susceptible. Hanxuan10 is a drought tolerant cultivar and Chadianhong is known to be salt tolerant, both of which are susceptible to the Bgt race No. 15. Wheat seedlings were grown in a chamber at 22°C and a photoperiod of 16 h (60 µmol m^−2^ s^−1^ photon flux density). For H_2_O_2_ treatment, 9-day-old seedlings were transferred to a Hoagland's solution containing 10 mM H_2_O_2_. For PM inoculation, the *Bgt* isolate E09 conidia on heavily diseased leaves were shaken off over a settling tower onto the wheat primary leaves, harvested at 0 and 6 h after treatment, and stored at −80°C until use. Inoculated plants were checked later to ensure proper development of powdery mildew on the leaves of the susceptible control.

### Measurements of Photosynthesis Efficiency and H_2_O_2_ Levels

Net photosynthetic rates of the first and second leaves of wheat seedlings were measured using a LI-COR 6400 portable gas analysis system with a light-emitting diode light source (LI-COR Inc., Lincoln, NE), with seven duplicates. H_2_O_2_ accumulation in wheat leaves was measured according to a previously described method [73]. Briefly, leaves were grounded to a fine powder and extracted with 5 mM titanium sulfate. The oxidation of titanium sulfate was recorded by reading the absorbance at 410 nm. The readings were converted to corresponding concentrations using a standard calibration plot.

### Tag Library Construction and the Initial Bioinformatic Processing

Wheat seedling leaves at 0 h and 6 h after H_2_O_2_ treatment were used for mRNA tag library construction, as described by [74]. Sequencing was performed by Beijing Genomics Institute, Shenzhen, China (http://sequencing.genomics.com.cn/). For the tag number counting, a reference tag database was generated using 274,754 PlantGDB sequences (Release 163b). For convenience, the prefix “PUT-163b-Triticum_aestivum” was replaced by a shorter one, “Ta”. A total of 217,691 sequences were found to have GATC sites, which generated a total of 425,312 reference tags, with340,097 unambiguous tags (79.96%). The tags derived from wheat mRNA libraries were counted for redundancy and, therefore, copy numbers, using in-house Perl scripts. Numbers of all the tags on one EST or assembly were used to represent its expression levels.

### mRNA Tag Data Analysis

Tag numbers for each gene were normalized with the total number of tags in the corresponding library. The statistical difference between corresponding genes in differently libraries was measured using a previously described method [75]. Expression level changes were calculated using the log2 ratios of transcripts per million mapped reads (TPM) between conditions (P6/PK, H6/HK, C6/CK). The sets of genes were selected for further analysis after the following filters: (1) TPM log2 ratios were either ≥1 for up regulation or ≤1 for down regulation; (2) the copy numbers for each condition should be ≥12; (3) the FDR for differential expression was set to be <0.001. For convenience, transcripts with zero detected tags in one condition were arbitrarily designated as 0.01 TPM and marked, whereas others were normalized using the total tag numbers in one library (minimum 12 copies for tags to be included for analysis). For MapMan analysis, wheat EST sequences were compared using Blastx against Arabidopsis sequences represented on Affymetrix GeneChips. Fold change numbers were then transferred to the best Arabidopsis matches which were mapped to the Arabidopsis template in the MapMan program for display [42]. The manual functional classification of genes followed Wan and Liu [15]. Gene Ontology enrichment analysis was performed using *Arabidopsis* proteins as templates at AGRIGO website (http://bioinfo.cau.edu.cn/agriGO/).

### Virus Induced Gene Silencing Assays

The plasmids utilized in these experiments were based on the constructs described by Holzberg *et al.* [76]. The virus-induced gene silencing of *TaFAD* (PlantGDB EST Ta-0109540) and *TaHSP90* (*TaHSP90.3-D1*, GQ240789.1; *TaHSP90.3-A1*, GQ240787.1) [51] was performed using a δ RNA vector, pSS031-1. *TaHSP90* was used as a positive control for powdery mildew resistance. A 231-bp fragment of *TaFAD* was amplified from the plasmid pTaFAD with the forward primer, CTAGCTAGCGGGGTCTTCTGGTACAGC, and the reverse primer, CTAGCTAGCGACACGCTACTCTTTCCTTT. A 355-bp fragment of *TaHSP90* was amplified from the plasmid pTaHSP90 with the forward primer, CTAGCTAGCGAGACCTTCGCCTTCCAG, and the reverse primer, CTAGCTAGCCACCGAACTGCCCAATCA. The underlined bases are the NheI restriction sites. Plants were infected with BSMV using a modified protocol [50], [76]. Briefly, capped transcripts were prepared from three linearized plasmids that contain the tripartite barley stripe mosaic virus (BSMV) genome using the mMessage mMachine T7 in vitro transcription kit (Ambion, Austin, TX), following the manufacturer's protocol. These in vitro transcription reactions typically resulted in a final concentration of 1 to 1.5 mg/ml of RNA.

### *Bgt*-wheat Interaction Assays

The method to estimate *Bgt* infection efficiency is largely in accord to Li *et al.* [11]. Wheat leaves of three centimeters were aligned on the surface of 0.5% agarose with 50 mg/L 2-[(4-chlorophenenyl)methyl]-1H-benzimidazol and sprayed with *Bgt* spores (isolate E09) using an air compressor and nozzle. After 5 days, the leaves were bleached with trichloroacetic acid (1.5 g L^−1^) in ethanol∶chloroform (4∶1 v/v), stained with aniline blue (1 g L^−1^), and observed under a light microscope for the formation of elongated secondary hyphae. A segment of the same leaf was kept at −80°C for mRNA extraction and subsequent quantitative real time PCR (qRT-PCR). For VIGS plants, newly emerging leaves of 14 d after the viral inoculation (usually the fourth leaves) were used. At least 8 VIGS plants were tested for each VIGS vector with three independent biological replicates.

### Measurements of Gene Expression Levels by qRT-PCR

For mRNA tag data and gene silencing confirmation, RNA was extracted using Trizol reagent (TIANGEN, China) and qRT-PCR experiments were performed on an ABI Prism® 7300 (Applied Biosystems, USA). The number of transcripts was normalized with the constitutively expressed glyceraldehyde-3-phosphate dehydrogenase (GAPDH) mRNA [77]–[78], which was tested as the most stable reference gene for the wheat seedling powdery mildew infection assay (data not shown).. The qRT-PCR assays were repeated three times, each with three biological replicates. To test the silencing efficiency, qRT-PCR primers were designed as: *TaFAD* forward, TACGTCGAGCCCGAGGACCG; *TaFAD* reverse, TGCCCCAAAATGCCCTCTTGCT; *TaFAD6* (Ta-1294165443) forward, GGGAGAAGTCACCACCAA; and *TaFAD6* reverse, GACCGAAAGCATACGAAG. RNA from the BSMV∶GFP-treated plants was used as controls.

## Supporting Information

Figure S1 MapMan biotic overview of PmA-specific H_2_O_2_ responding genes.
(TIF)

Table S1 The statistics of the mRNA tags from the six wheat libraries in this study.
(XLS)

Table S2 The statistics of the differentially expressed transcripts in response to H_2_O_2_ treatment in the wheat lines PmA, Han, and Cha.
(XLS)

Table S3 The correlation coefficients (*R^2^*) of the mRNA tag numbers for genes shared among different libraries.
(XLS)

Table S4 **q**RT-PCR validation of 28 differentially expressed genes as detected by the miRNA tag analysis.
(XLS)

Table S5 The full list of genes with the same differential expression patterns in all three wheat lines under H_2_O_2_ stress.
(XLS)

Table S6 Classification of H_2_O_2_ responsive genes with specific expression patterns in PmA.
(XLS)

Table S7 The full list of 2,982 PmA-specific differentially expressed ESTs under H_2_O_2_ treatment.
(XLS)

Table S8 Representative H_2_O_2_ regulated genes associated with the enriched GO term vesicle-mediated transport from the class I PmA specific genes.
(XLS)

Table S9 Functional categories of PmA-specific H_2_O_2_ responding genes from the MapMan biotic stress overview.
(XLS)

Table S10 Functional classification of 328 PmA-specific ESTs from the biotic overview in MapMan.
(XLS)

Table S11 qRT-PCR confirmation of JA/Et signal pathway genes under H_2_O_2_ treatment.
(XLS)

Table S12 *Blumeria graminis* f. sp. *tritici* penetration efficiency (PE) in the VIGS plants.
(XLS)

Table S13 Expression patterns of putative JA and SA signaling pathway related genes in the *BSMV∶FAD* and *BSMV∶HSP90* plants.
(XLS)

## References

[1] Costa A, Drago I, Behera S, Zottini M, Pizzo P (2010). H_2_O_2_ in plant peroxisomes: an *in vivo* analysis uncovers a Ca^2+^-dependent scavenging system.. Plant Journal.

[2] Prasad TK, Anderson MD, Martin BA, Stewart CR (1994). Evidence for chilling-induced oxidative stress in maize seedlings and a regulatory role for hydrogen peroxide.. Plant Cell.

[3] Loggini B, Scartazza A, Brugnoli E, Navari-Izzo F (1999). Antioxidative defense system, pigment composition, and photosynthetic efficiency in two wheat cultivars subjected to drought.. Plant Physiology.

[4] Valderrama R, Corpas FJ, Carreras A, Gomez-Rodriguez MV, Chaki M (2006). The dehydrogenase-mediated recycling of NADPH is a key antioxidant system against salt-induced oxidative stress in olive plants.. Plant, Cell & Environment.

[5] Mackerness S, Surplus SL, Blake P, John CF, Buchanan-Wollaston V (1999). Ultraviolet-B induced stress and changes in gene expression in Arabidopsis thaliana: role of signaling pathways controlled by jasmonic acid, ethylene and reactive oxygen species.. Plant, Cell & Environment.

[6] Pellinen R, Palva T, Kangasja J (1999). Subcellular localization of ozone-induced hydrogen peroxide production in birch (*Betula pendula*) leaf cells.. Plant Journal.

[7] Schutzendubel A, Polle A (2002). Plant responses to abiotic stresses: heavy metal-induced oxidative stress and protection by mycorrhization.. Journal of Experimental Botany.

[8] Liu Y, Huang W, Zhan J, Pan Q (2005). Systemic induction of H_2_O_2_ in pea seedlings pretreated by wounding and exogenous jasmonic acid.. Science in China Series C: Life Sciences.

[9] Pei ZM, Murata Y, Benning G, Thomine S, Klusener B (2000). Calcium channels activated by hydrogen peroxide mediate abscisic acid signalling in guard cells.. Nature.

[10] Levine A, Tenhaken R, Dixon R, Lamb C (1994). H_2_O_2_ from the oxidative burst orchestrates the plant hypersensitive disease resistance response.. Cell.

[11] Li A, Wang M, Zhou R, Kong X, Huo N (2005). Comparative analysis of early H_2_O_2_ accumulation in compatible and incompatible wheat-powdery mildew interactions.. Plant Pathology.

[12] Desikan R, Mackerness S, Hancock JT, Neill SJ (2001). Regulation of the Arabidopsis transcriptome by oxidative stress.. Plant Physiology.

[13] Mittler R, Vanderauwera S, Gollery M, Van Breusegem F (2004). Reactive oxygen gene network of plants.. Trends in Plant Science.

[14] Vanderauwera S, Zimmermann P, Rombauts S, Vandenabeele S, Langebartels C (2005). Genome-wide analysis of hydrogen peroxide-regulated gene expression in Arabidopsis reveals a high light-induced transcriptional cluster involved in anthocyanin biosynthesis.. Plant Physiology.

[15] Wan X, Liu J (2008). Comparative proteomics analysis reveals an intimate protein network provoked by hydrogen peroxide stress in rice seedling leaves.. Molecular & Cell Proteomics.

[16] Lenucci MS, Piro G, Dalessandro G (2009). In muro feruloylation and oxidative coupling in monocots A possible role in plant defense against pathogen attacks.. Plant Signaling & Behaviour.

[17] Kovtun Y, Chiu WL, Tena G, Sheen J (2000). Functional analysis of oxidative stress-activated mitogen-activated protein kinase cascade in plants.. Proceedings of the National Academy of Sciences, USA.

[18] Mou Z, Fan W, Dong X (2003). Inducers of plant systemic acquired resistance regulate NPR1 function through redox changes.. Cell.

[19] Montillet JL, Chamnongpol S, Rusterucci C, Dat J, van de Cotte B (2005). Fatty acid hydroperoxides and H_2_O_2_ in the execution of hypersensitive cell death in tobacco leaves.. Plant Physiology.

[20] Thoma I, Loeffler C, Sinha AK, Gupta M, Krischke M (2003). Cyclopentenone isoprostanes induced by reactive oxygen species trigger defense gene activation and phytoalexin accumulation in plants.. Plant Journal.

[21] Mur LAJ, Kenton P, Lloyd AJ, Ougham H, Prats E (2008). The hypersensitive response; the centenary is upon us but how much do we know?. Journal of Experimental Botany.

[22] Torres MA, Jones JDG, Dangl JL (2006). Reactive oxygen species signaling in response to pathogens.. Plant Physiology.

[23] Blee E (2002). Impact of phyto-oxylipins in plant defense.. Trends in Plant Science.

[24] Kunkel B, Brooks D (2002). Cross talk between signaling pathways in pathogen defense.. Current Opinion in Plant Biology.

[25] Overmyer K, Brosche M, Kangasjarvi J (2003). Reactive oxygen species and hormonal control of cell death.. Trends in Plant Science.

[26] Bouchez O, Huard C, Lorrain S, Roby D, Balague C (2007). Ethylene is one of the key elements for cell death and defense response control in the Arabidopsis lesion mimic mutant vad1.. Plant Physiology.

[27] Vanacker H, Carver T, Foyer C (2000). Early H_2_O_2_ accumulation in mesophyll cells leads to induction of glutathione during the hyper-sensitive response in the barley-powdery mildew interaction.. Plant Physiology.

[28] Torres MA (2010). ROS in biotic interactions.. Physiologia Plantarum.

[29] Despres C, Chubak C, Rochon A, Clark R, Bethune T (2003). The Arabidopsis NPR1 disease resistance protein is a novel cofactor that confers redox regulation of DNA binding activity to the basic domain/leucine zipper transcription factor TGA1.. Plant Cell.

[30] Cao AZ, Xing LP, Wang XY, Yang XM, Wang W (2011). Serine/threonine kinase gene Stpk-V, a key member of powdery mildew resistance gene Pm21, confers powdery mildew resistance in wheat.. Proceedings of National Academy of Sciences, USA.

[31] Anthony RG, Henriques R, Helfer A, Meszaros T, Rios G (2004). A protein kinase target of a PDK1 signalling pathway is involved in root hair growth in Arabidopsis.. EMBO Journal.

[32] Stone JR (2004). An assessment of proposed mechanisms for sensing hydrogen peroxide in mammalian systems.. Archives of Biochemistry and Biophysics.

[33] Qin D, Wu H, Peng H, Yao Y, Ni Z (2008). Heat stress-responsive transcriptome analysis in heat susceptible and tolerant wheat (*Triticum aestivum* L.) by using Wheat Genome Array.. BMC Genomics.

[34] Aprile A, Mastrangelo A, De Leonardis A, Galiba G, Roncaglia E (2009). Transcriptional profiling in response to terminal drought stress reveals differential responses along the wheat genome.. BMC Genomics.

[35] Hulbert S, Bai J, Fellers J, Pacheco M, Bowden R (2007). Gene expression patterns in near isogenic lines for wheat rust resistance gene Lr34/Yr18.. Phytopathology.

[36] Coram T, Settles M, Chen X (2008). Transcriptome analysis of high-temperature adult-plant resistance conditioned by Yr39 during the wheat-Puccinia striiformis f. sp. tritici interaction.. Molecular Plant Pathology.

[37] Boyd L (2009). Wheat blast: histopathology and transcriptome reprogramming in response to adapted and nonadapted Magnaporthe isolates.. New Phytologist.

[38] Desmond OJ, Manners JM, Schenk PM, Maclean DJ, Kazan K (2008). Gene expression in the wheat response to infection by *Fusarium pseudograminearum*.. Physiological and Molecular Plant Pathology.

[39] Chain F, Cote-Beaulieu C, Belzile F, Menzies J, Belanger R (2009). A Comprehensive Transcriptomic Analysis of the Effect of Silicon on Wheat Plants Under Control and Pathogen Stress Conditions.. Molecular Plant-Microbe Interaction.

[40] Schreiber A, Sutton T, Caldo R, Kalashyan E, Lovell B (2009). Comparative transcriptomics in the Triticeae.. BMC Genomics.

[41] An QL, Ehlers K, Kogel KH, van Bel AJE, Hückelhoven R (2006). Multivesicular compartments proliferate in susceptible and resistant MLA12-barley leaves in response to infection by the biotrophic powdery mildew fungus.. New Phytologist.

[42] Thimm O, Blsing O, Gibon Y, Nagel A, Meyer S (2004). MAPMAN: A user-driven tool to display genomics data sets onto diagrams of metabolic pathways and other biological processes.. Plant Journal.

[43] Zeng LR, Vega-Sánchez ME, Zhu T, Wang GL (2006). Ubiquitination-mediated protein degradation and modification: an emerging theme in plant-microbe interactions.. Cell Research.

[44] Romeis T (2001). Protein kinase in the plant defence response.. Current Opinion in Plant Biology.

[45] McConn M, Creelman R, Bell E, Mullet J (1997). Jasmonate is essential for insect defense in Arabidopsis.. Proceedings of the National Academy of Sciences, USA.

[46] Vijayan P, Shockey J, Levesque C, Cook R (1998). A role for jasmonate in pathogen defense of Arabidopsis.. Proceedings of the National Academy of Sciences, USA.

[47] Thomma B, Eggermont K, Broekaert W, Cammue B (2000). Disease development of several fungi on Arabidopsis can be reduced by treatment with methyl jasmonate.. Plant Physiology and Biochemistry.

[48] Kachroo P, Shanklin J, Shah J, Whittle E, Klessig D (2001). A fatty acid desaturase modulates the activation of defense signaling pathways in plants.. Proceedings of National Academy of Sciences, USA.

[49] Jiang C, Shimono M, Maeda S, Inoue H, Mori M (2009). Suppression of the rice fatty-acid desaturase gene OsSSI2 enhances resistance to blast and leaf blight diseases in rice.. Molecular Plant-Microbe Interaction.

[50] Scofield S, Huang L, Brandt A, Gill B (2005). Development of a virus-induced gene-silencing system for hexaploid wheat and its use in functional analysis of the Lr21-mediated leaf rust resistance pathway.. Plant Physiology.

[51] Wang GF, Wei XN, Fan RC, Zhou HB, Wang XP (2011). Molecular analysis of common wheat genes encoding three types of cytosolic heat shock protein 90 (Hsp90): functional involvement of cytosolic Hsp90s in the control of wheat seedling growth and disease resistance.. New Phytologist.

[52] Qiu D, Xiao J, Ding X, Xiong M, Cai M (2007). OsWRKY13 mediates rice disease resistance by regulating defense-related genes in salicylate- and jasmonate-dependent signaling.. Molecular Plant-Microbe Interaction.

[53] Tao Z, Liu H, Qiu D, Zhou Y, Li X (2009). A pair of allelic WRKY genes play opposite role in rice-bacteria interactions.. Plant Physiology.

[54] Lu R, Malcuit I, Moffett P, Ruiz MT, Peart J (2003). High throughput virus-induced gene silencing implicates heat shock protein 90 in plant disease resistance.. EMBO Journal.

[55] Hein I, Barciszewska-Pacak M, Hrubikova K, Williamson S, Dinesen M (2005). Virus-induced gene silencingbased functional characterization of genes associated with powdery mildew resistance in barley.. Plant Physiology.

[56] Zhou HB, Li SF, Deng ZY, Wang XP, Chen T, Zhang JS (2007). Molecular analysis of three new receptor-like kinase genes from hexaploid wheat and evidence for their participation in the wheat hypersensitive response to stripe rust fungus infection.. Plant Journal.

[57] Matsuda Y, Okuda T, Sagisaka S (1994). Regulation of Protein Synthesis by Hydrogen Peroxide in Crowns of Winter Wheat.. Bioscience,Biotechnology, and Biochemistry.

[58] Collins NC, Thordal-Christensen H, Lipka V, Bau S, Kombrink E (2003). SNARE-protein-mediated disease resistance at the plant cell wall.. Nature.

[59] Leshem Y, Melamed–Book, Cagnac O, Ronen G, Nishri Y (2006). Suppression of Arabidopsis vesicle-SNARE expression inhibited fusion of H_2_O_2_-containing vesicles with tonoplast and increased salt tolerance.. Proceedings of National Academy of Sciences, USA.

[60] Fryer MJ, Ball L, Oxborough K, Karpinski S, Mullineaux PM (2003). Control of ascorbate peroxidase 2 expression by hydrogen peroxide and leaf water status during excess light stress reveals a functional organisation of Arabidopsis leaves.. Plant Journal.

[61] Chang CCC, Ball L, Fryer MJ, Baker NR, Karpinski S (2004). Induction of ascorbate peroxidase 2 expression in wounded Arabidopsis leaves does not involve known wound-signalling pathways but is associated with changes in photosynthesis.. Plant Journal.

[62] Mateo A, Muhlenbock P, Rusterucci C, Chang CCC, Miszalski Z (2004). Lesion simulating disease 1 is required for acclimation to conditions that promote excess excitation energy.. Plant Physiology.

[63] Overmyer K, Brosche M, Pellinen R, Kuittenen T, Tuominen H (2005). Ozone-induced programmed cell death in the Arabidopsis radical-induced cell death 1 mutant.. Plant Physiology.

[64] Mateo A, Funck D, Muhlenbock P, Kular B, Mullineaux PM (2006). Controlled levels of salicylic acid are required for optimal photosynthesis and redox homeostasis.. Journal of Experimental Botany.

[65] Muhlenbock P, Plaszczyca M, Plaszczyca M, Mellerowicz E, Karpinski S (2007). Lysigenous aerenchyma formation in Arabidopsis is controlled by lesion simulating disease1.. Plant Cell.

[66] Laudert D, Weiler EW (1998). Allene oxide synthase: A major control point in Arabidopsis thaliana octadecanoid signalling.. Plant Journal.

[67] Kachroo A, Lapchyk L, Fukushigae H, Hildebrand D, Klessig D (2003a). Plastidal fatty acid signaling modulates SA- and JA-mediated signaling in the Arabidopsis ssi2 mutant.. Plant Cell.

[68] Kachroo P, Kachroo A, Lapchyk L, Hildebrand D, Klessig D (2003b). Restoration of defective cross talk in *ssi2* mutant: Role of salicylic acid, jasmonic acid, and fatty acids in *SSI2*-mediated signaling.. Molecular Plant-Microbe Interaction.

[69] Kachroo P, Srivathsa CV, Navarre DA, Lapchyk L, Kachroo A (2005). Role of salicylic acid and fatty acid desaturation pathways in *ssi2*-mediated signaling.. Plant Physiology.

[70] Kachroo A, Daqi F, Havens W, Navarre R, Kachroo P (2008). Virus-induced gene silencing of stearoyl-acyl carrier protein-desaturase in soybean results in constitutive defense and enhanced resistance to pathogens.. Molecular Plant-Microbe Interaction.

[71] Chaturvedi R, Krothapalli K, Makandar R, Nandi A, Sparks AA (2008). Plastid ω3-fatty acid desaturase-dependent accumulation of a systemic acquired resistance inducing activity in petiole exudates of Arabidopsis thaliana is independent of jasmonic acid.. Plant Journal.

[72] Zhou RH, Zhu ZD, Kong XY, Huo NX, Tian QZ (2005). Development of wheat near-isogenic lines for powdery mildew resistance.. Theoretical and Applied Genetics.

[73] Tiwari B, Belenghi B, Levine A (2002). Oxidative stress increased respiration and generation of reactive oxygen species, resulting in ATP depletion, opening of mitochondrial permeability transition, and programmed cell death.. Plant Physiology.

[74] Hoen P, Ariyurek Y, Thygesen H, Vreugdenhil E, Vossen R (2008). Deep sequencing-based expression analysis shows major advances in robustness, resolution and inter-lab portability over five microarray platforms.. Nucleic Acids Research.

[75] Audic S, Claverie J (1997). The significance of digital gene expression profiles.. Genome Research.

[76] Holzberg S, Brosio P, Gross C, Pogue G (2002). Barley stripe mosaic virus-induced gene silencing in a monocot plant.. Plant Journal.

[77] Hong S, Seo P, Yang M, Xiang F, Park C (2008). Exploring valid reference genes for gene expression studies in Brachypodium distachyon by real-time PCR.. BMC Plant Biology.

[78] Paolacci AR, Tanzarella OA, Porceddu E, Ciaffi M (2009). Identification and validation of reference genes for quantitative RT-PCR normalization in wheat.. BMC Molecular Biology.

