# Supplementary figures and images for: Transcriptome Analysis of H2O2-Treated Wheat Seedlings Reveals a H2O2-Responsive Fatty Acid Desaturase Gene Participating in Powdery Mildew Resistance
Source: PLoS One. 2011 Dec 12;6(12):e28810. doi: 10.1371/journal.pone.0028810 (PMC3236209; doi:10.1371/journal.pone.0028810)

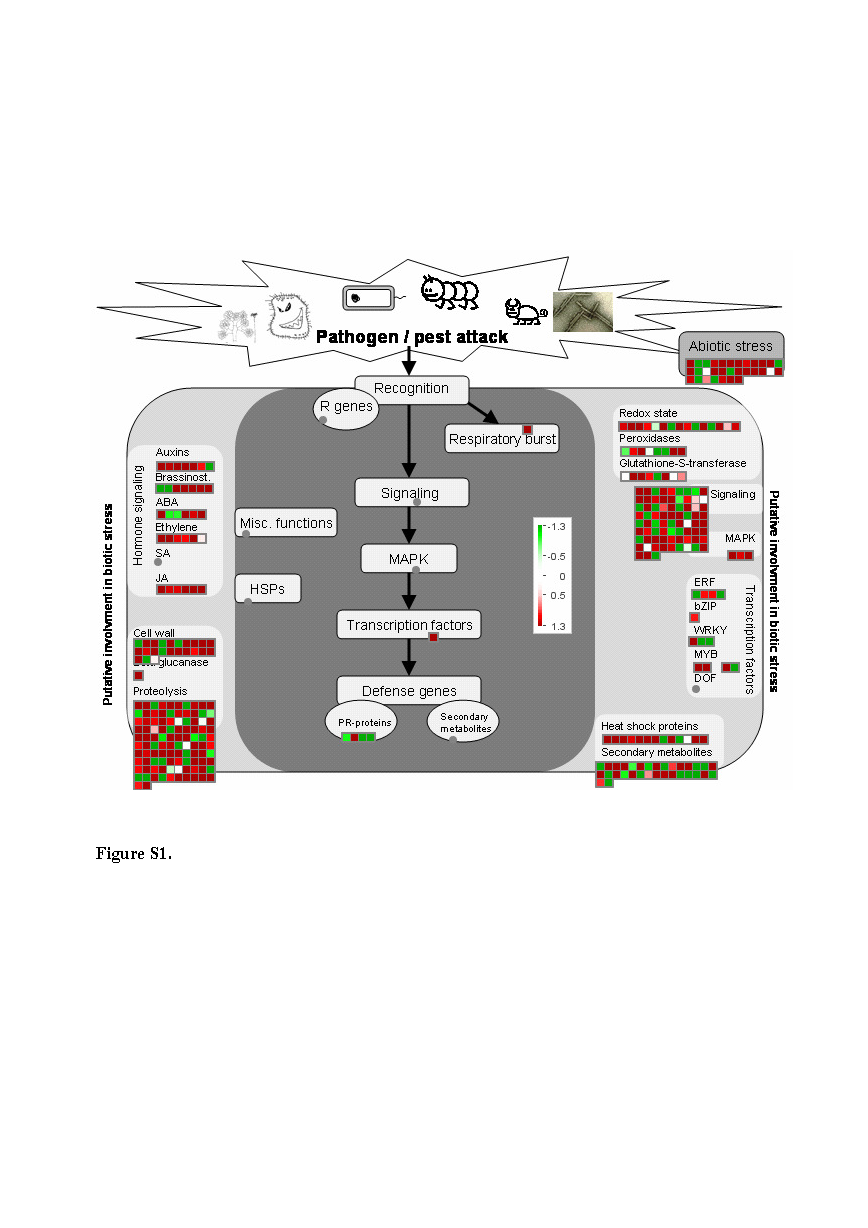

Supplement: Figure S1 — MapMan biotic overview of PmA-specific H2O2 responding genes. (TIF) [file pone.0028810.s001.tif]
